# Rapamycin protects chondrocytes against IL-18-induced apoptosis and ameliorates rat osteoarthritis

**DOI:** 10.18632/aging.102937

**Published:** 2020-03-17

**Authors:** Jiapeng Bao, Zhonggai Chen, Langhai Xu, Lidong Wu, Yan Xiong

**Affiliations:** 1 Department of Orthopedic Surgery, The Second Affiliated Hospital, School of Medicine, Zhejiang University, Hangzhou 310000, Zhejiang, China

**Keywords:** interleukin 18, osteoarthritis, chondrocyte apoptosis, rapamycin, mTOR

## Abstract

Interleukin 18 (IL-18) promotes inflammation and apoptosis in chondrocytes, thereby contributing to the development and progression of osteoarthritis (OA). Here, we investigated the effects of IL-18 treatment and inhibition in rat chondrocytes *in vitro* and *in vivo.* We used RT-PCR and Western blotting to measure the mRNA and protein levels of the chondrocyte-specific genes Collagen II and Aggrecan as well as the protein levels of apoptosis-related (Bax, Bcl2, Caspase3/9), autophagy-related (Atg5, Atg7, Beclin1, LC3), and mTOR pathway-related genes (PI3K, Akt, mTOR). We observed a decrease in Collagen II and Aggrecan mRNA and protein levels, upregulation of chondrocyte apoptosis, downregulation of chondrocyte autophagy, and activation of the PI3K/Akt/mTOR pathway upon IL-18 treatment. PI3K/Akt/mTOR pathway activation and inhibition tests using rat 740Y-P (PI3K activator), SC79 (AKT activator), 3BDO (mTOR activator), or LY294002 (PI3K inhibitor) revealed that activation of the PI3K/Akt/mTOR pathway enhances chondrocyte-specific gene degradation induced by IL-18, while its inhibition has protective effects on chondrocytes. We also found that treatment with rapamycin (a selective mTOR inhibitor) also exerts chondro-protective effects that ameliorate OA by promoting autophagy. These results suggest that inhibition of the mTOR pathway could be exploited for therapeutic benefits in the treatment of OA.

## INTRODUCTION

Osteoarthritis (OA) is the most frequent form of arthritis and a leading cause of pain and disability among the elderly worldwide [1]. There are various causes for OA, including aging, obesity, and trauma, among others [2]. The main strategy to manage early OA consists of administering anti-inflammation drugs to patients. In spite of the development of new anti-inflammation drugs [3–6], OA remains incurable and the condition often worsen over time. The pathological changes implied in OA progression impact all the tissues forming a joint and are primarily characterized by cartilage degradation. Cartilage is composed of chondrocytes and their extracellular matrix, which is rich in collagens and proteoglycans. Recent research suggests that the development and progression of OA and the associated degradation of cartilage are induced by immune system cytokines [7, 8]. Indeed, tumor necrosis factor α (TNFα) and Interleukin 1β (IL1β) promote cartilage degradation in OA models [9–11].

Interleukin 18 (IL-18) is a member of the IL-1 family [12] and is produced by chondrocytes, osteoblasts, and macrophages in joints [13, 14]. It induces inflammatory responses in synoviocytes and chondrocytes [15], is elevated in the synovial fluid of patients with OA [16], promotes the release of metalloproteinases MMP-1, MMP-3, and MMP-13 from chondrocytes [17], and increases the production of pro-inflammatory factors, such as IL6, iNOS, COX2, and PGE2 [13, 18, 19]. While apoptosis and autophagy are known to be involved in cartilage degradation, the specific effects of IL-18 on chondrocyte apoptosis and autophagy remain unclear. IL-18 may contribute to chondrocyte apoptosis by inhibition of MAPK signaling [20]; however, the detailed underlying mechanisms are poorly understood. Thus, here we investigated the effects of IL-18 and rapamycin (a selective inhibitor of the mTOR pathway) treatments on chondrocyte apoptosis and autophagy *in vitro* and *in vivo*.

## RESULTS

### Chondrocyte-specific genes degradation is caused by IL-18 stimulation in rat chondrocytes *in vitro*

**Figure 1:**
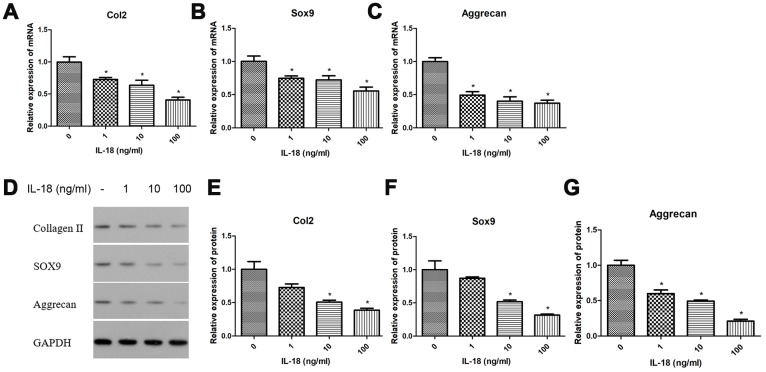
**Chondrocyte-specific genes degradation caused by IL-18 was observed in rat chondrocytes.** Messenger RNA expression of chondrocyte-specific genes, Collagen II (**A**), Sox9 (**B**), and Aggrecan (**C**) evaluated by Real-Time PCR, and expression of chondrocyte-specific proteins, collagen II (**E**), sox9 (**F**), aggrecan (**G**), and GAPDH as an internal control evaluated by Western blot (**D**) in chondrocytes treated with IL-18 at different concentrations for 24 h. The values are expressed as mean ± standard deviation (SD). Significance was calculated by a one-way ANOVA with a *post hoc* Tukey's multiple comparisons test. *p<0.05 versus 0ng/ml IL-18 treated group.

We investigated the effects of IL-18 on chondrocyte-specific gene expression in rat chondrocytes using RT-PCR and Western blot. Chondrocytes were treated with IL-18 at different concentrations (0, 1, 10, 100 ng/ml) for 24 h, then the mRNA expression and protein levels of Collagen II, Sox9, and Aggrecan were evaluated by RT-PCR and Western blot, respectively. As shown in Figure 1, the mRNA expression of Collagen II, Sox9 and Aggrecan was downregulated by IL-18 treatment, which is IL-18 dose-dependent. Similarly, the protein levels of collagen II, sox9 and aggrecan showed the same tendency after IL-18 treatment. All these results suggested that IL-18 promotes chondrocyte-specific gene degradation by downregulating Collagen II, Sox9 and Aggrecan in a dose-dependent manner *in vitro*.

### IL-18 stimulation induced chondrocyte apoptosis *in vitro*

**Figure 2:**
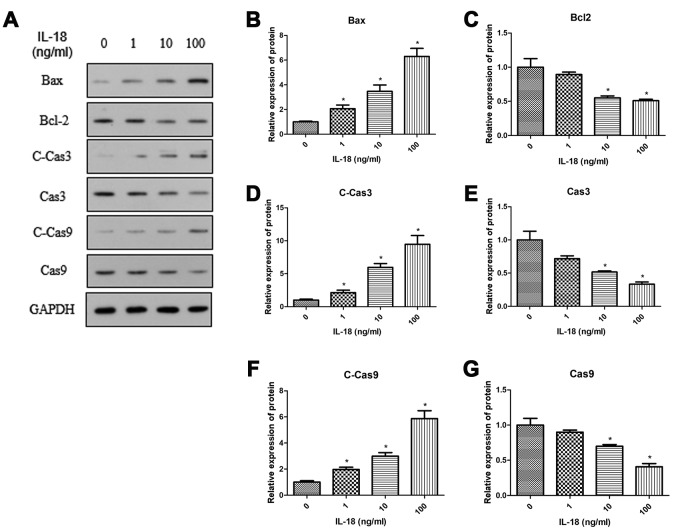
**IL-18 stimulation increased the protein levels of pro-apoptosis genes and decreased the protein levels of anti-apoptosis genes.** Western blot (**A**) measuring protein levels of Bax (**B**), Bcl2 (**C**), Cleaved Caspase3 (**D**), Caspase3 (**E**), Cleaved Caspase9 (**F**), Caspase9 (**G**), and GAPDH as an internal control in total extract from chondrocytes treated with IL-18 at different concentrations for 24 h. The values are expressed as mean ± standard deviation (SD). Significance was calculated by a one-way ANOVA with a *post hoc* Tukey's multiple comparisons test. *p<0.05 versus 0ng/ml IL-18 treated group.

Chondrocyte apoptosis contributes to the development of OA. We performed Western blot experiments to reveal the effect of IL-18 on apoptosis in chondrocytes. Chondrocytes were treated with IL-18 at different concentrations (0, 1, 10, 100 ng/ml) for 24 h, then the protein levels of Bax, Bcl2, and activation of Caspase3/9 were quantitated by Western blot. The results showed that IL-18 stimulation increased the expression of pro-apoptotic protein Bax and decreased the expression of anti-apoptotic protein Bcl2 while activating Caspase3/9, which were all IL-18 dose-dependent (Figure 2). This indicated that IL-18 induces apoptosis of chondrocytes *in vitro*.

### IL-18 stimulation induced chondrocyte senescence *in vitro*

Chondrocyte senescence also promotes OA progression. To assess the effect of IL-18 on chondrocyte senescence, we calculated the percentage of SA-β-Gal positive cells after incubating chondrocytes with different concentrations of IL-18 (0, 1, 10, 100 ng/ml) for 24 h. The results showed that IL-18 treatment increased the percentage of SA-β-Gal positive cells, and that IL-18 had a dose-dependent effect on chondrocyte senescence (Supplementary Figure 1).

### IL-18 stimulation induced autophagy deficiency of chondrocytes *in vitro*

**Figure 3:**
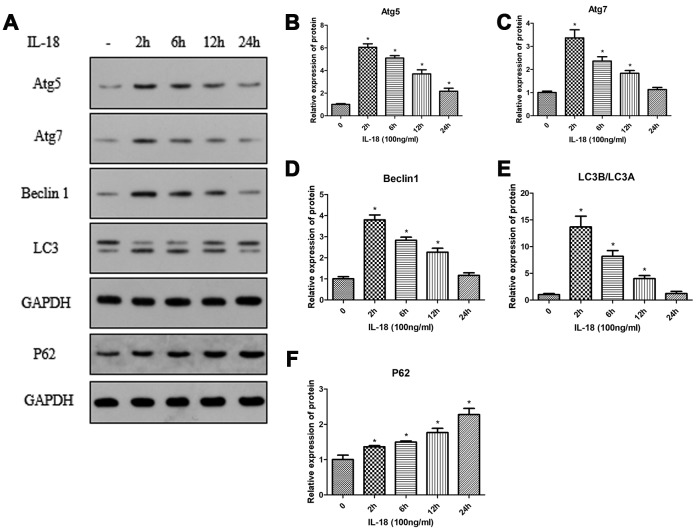
**IL-18 stimulation induced autophagy deficiency of chondrocytes *in vitro*.** Protein levels of Atg5 (**B**), Atg7 (**C**), Beclin1 (**D**), LC3B/LC3A (**E**), P62 (**F**), and GAPDH as an internal control in chondrocytes incubated without or with IL-18 (100 ng/ml) for various durations as evaluated by Western blot (**A**). The values are expressed as mean ± standard deviation (SD). Significance was calculated by a one-way ANOVA with a *post hoc* Tukey's multiple comparisons test. *p<0.05 versus without IL-18 treated group.

Autophagy plays an important role in cell growth and development and helps to maintain a balance between the anabolism and catabolism in chondrocytes. Here, we assessed autophagy activity using Western blot and immunofluorescence to determine whether defects in autophagy are associated with IL-18-induced chondrocyte apoptosis and senescence. We found that the protein levels of Atg5, Atg7, Beclin1, and the LC3B/LC3A ratio were upregulated after IL-18 (100 ng/ml) treatment for 2 h, and then decreased over time (Figure 3). Consistent with these results, immunofluorescence experiments also showed increased LC3B fluorescence intensity in chondrocytes after IL-18 treatment (100 ng/ml) for 6 h, and then decreased after 24 h (Supplementary Figure 2). P62/SQSTM1 functions as a selective autophagy receptor for degradation of ubiquitinated substrates, and the accumulation of P62 represents repressed autophagic degradation [21]. In this study, the level of P62 was increased under the IL-18 treatment within 24 h (Figure 3), implying that IL-18 stimulation induced autophagy deficiency of chondrocytes.

### Rapamycin pre-treatment rescued autophagy and decreased chondrocyte apoptosis *in vitro*

**Figure 4:**
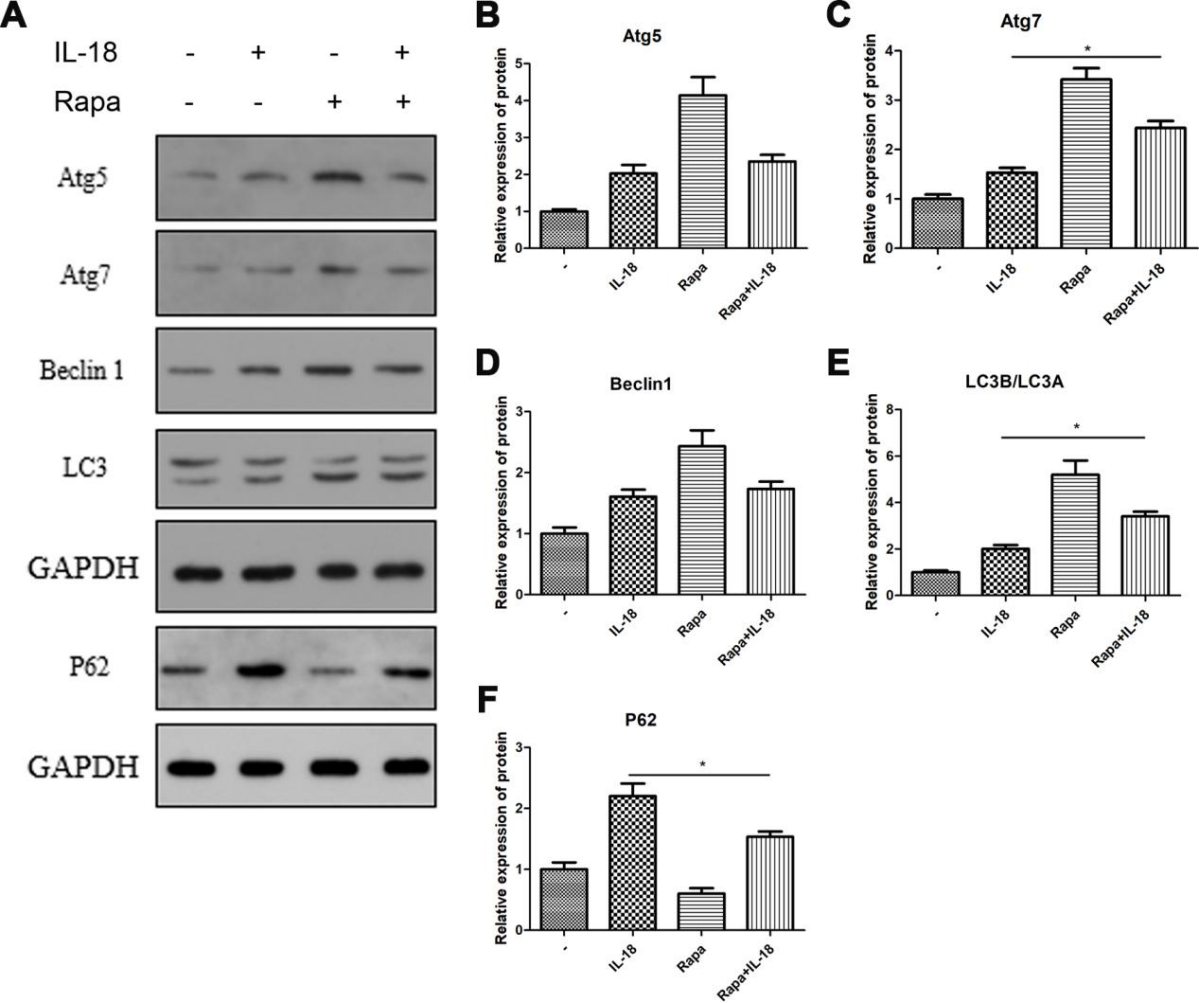
**Rapamycin protected chondrocytes against the autophagy deficiency caused by IL-18 stimulation.** Chondrocytes of the IL-18 + rapamycin treatment group were pre-treated with rapamycin (100 nM) for 1 h, followed with 24 h IL-18 stimulation (100 ng/ml). Chondrocytes of the IL-18 treatment group were treated with 100 ng/ml IL-18 for 24 h. Chondrocytes of the rapamycin treatment group were treated with 100 nM rapamycin for 24 h. Protein levels of Atg5 (**B**), Atg7 (**C**), Beclin1 (**D**), LC3B/LC3A (**E**), P62 (**F**), and GAPDH as an internal control, evaluated by Western blot (**A**). The values are expressed as mean ± standard deviation (SD). Significance was calculated by a one-way ANOVA with a *post hoc* Tukey's multiple comparisons test. *p<0.05 versus the IL-18 treatment group.

**Figure 5:**
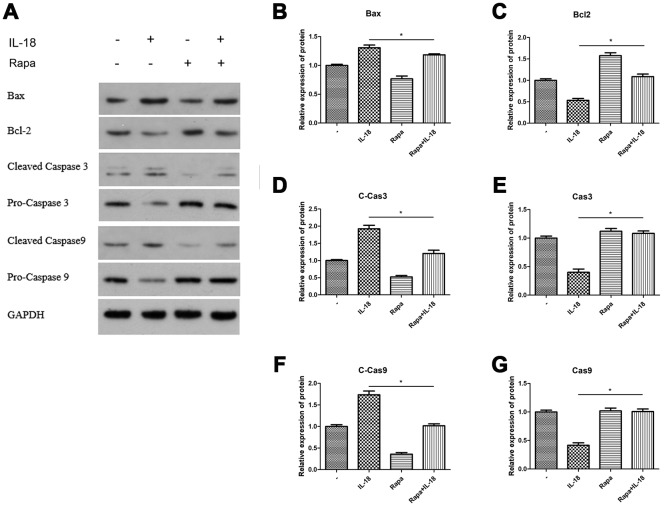
**Rapamycin worked as autophagy agonist and the anti-apoptosis effect of rapamycin was analyzed *in vitro*.** Chondrocytes of the IL-18 + rapamycin treatment group were pre-treated with rapamycin (100 nM) for 1 h, followed with 24 h IL-18 stimulation (100 ng/ml). Chondrocytes of the IL-18 treatment group were treated with 100 ng/ml IL-18 for 24 h. Chondrocytes of the rapamycin treatment group were treated with 100 nM rapamycin for 24 h. Protein levels of Bax (**B**) Bcl2 (**C**) Cleaved-Caspase3 (**D**) Caspase3 (**E**) Cleaved Caspase9 (**F**) Caspase9 (**G**) and GAPDH as an internal control, evaluated by Western blot (**A**). The values are expressed as mean ± standard deviation (SD). Significance was calculated by a one-way ANOVA with a *post hoc* Tukey's multiple comparisons test. *p<0.05 versus the IL-18 treatment group.

We used rapamycin (a commonly used autophagy agonist) to test whether IL-18-induced chondrocyte apoptosis could be suppressed by recovering the suppressed autophagy caused by IL-18. Chondrocytes were pretreated with rapamycin (100 nM) for 1 h before IL-18 treatment for 24 h, and then autophagy and apoptosis activities were measured by Western blot. The results showed that rapamycin pre-treatment increased the protein levels of Atg7 and the LC3B/LC3A ratio while decreasing P62 levels (Figure 4), which revealed that rapamycin could recover the suppressed activity of autophagy by IL-18 stimulation. What’s more, rapamycin pre-treatment efficiently protected against IL-18-induced apoptosis. As shown in Figure 5, the expression of the pro-apoptotic protein Bax and activation of caspase3/9 was reduced while that of of the anti-apoptotic protein Bcl2 was enhanced in rapamycin-pretreated chondrocytes. Thus, we demonstrated that rapamycin regulates IL-18-induced chondrocyte apoptosis by repairing the autophagy deficiency caused by IL-18 stimulation.

### IL-18 stimulation induced autophagy deficiency via the PI3K/Akt/mTOR signaling pathway

**Figure 6:**
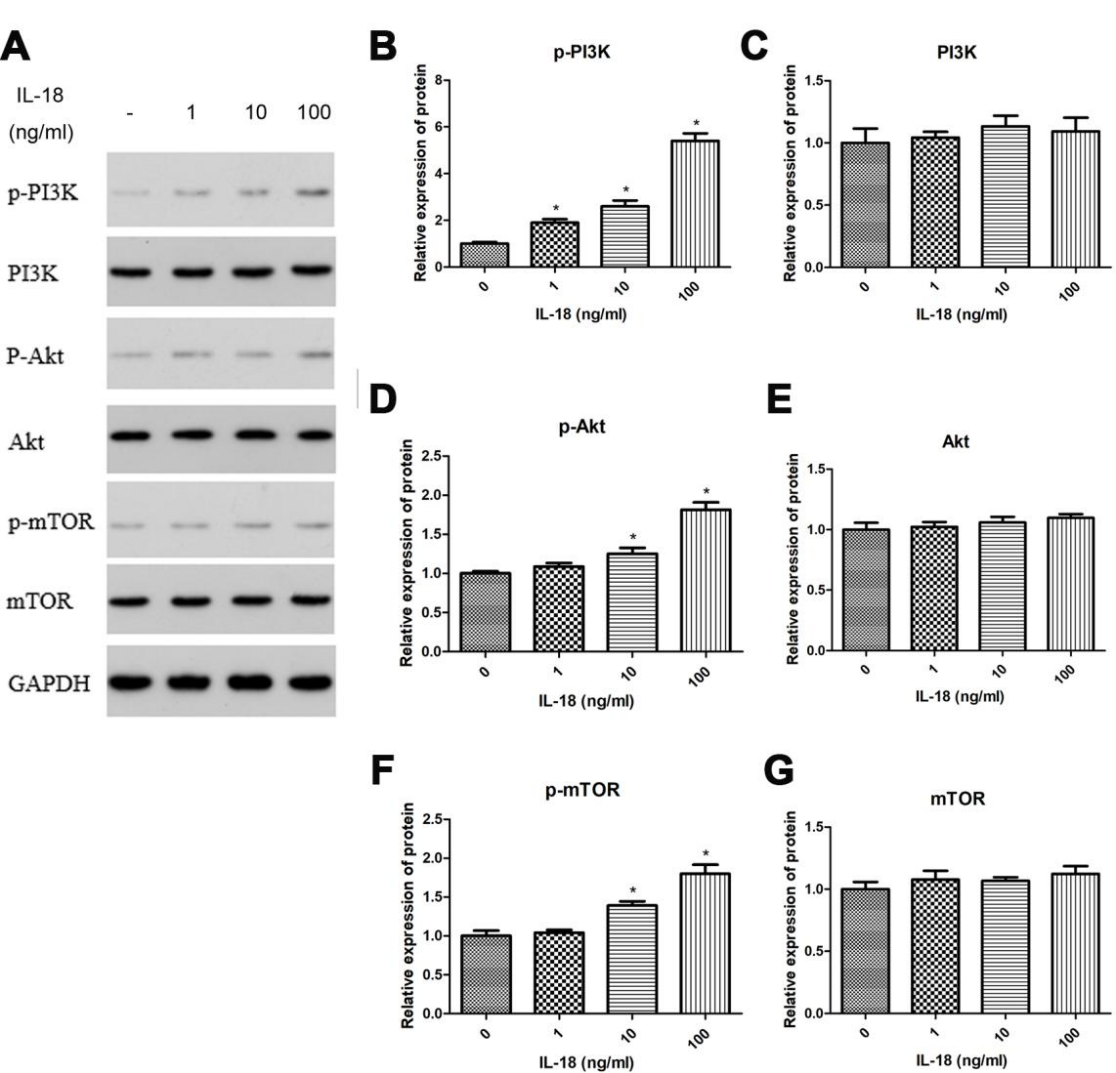
**IL-18 stimulation induced autophagy deficiency via PI3K/Akt/mTOR signaling pathway.** The chondrocytes were treated with IL-18 at different concentrations for 24 h. Protein levels of p-PI3K (**B**), PI3K (**C**), p-Akt (**D**), Akt (**E**), p-mTOR (**F**), mTOR (**G**), and GAPDH as an internal control in total extract, analyzed by Western blot (**A**). The values are expressed as mean ± standard deviation (SD). Significance was calculated by a one-way ANOVA with a *post hoc* Tukey's multiple comparisons test. *p<0.05 versus 0 ng/ml IL-18 treated group.

Mammalian target of rapamycin (mTOR) is a specific binding partner of rapamycin, and the mTOR signaling pathway promotes autophagic cell death [22]. To test whether IL-18 induced autophagy deficiency via the mTOR pathway, we treated chondrocytes with IL-18 at different concentrations (0, 1, 10, 100 ng/ml) for 24 h and measured the amounts of various proteins in whole cells and nuclei, separately. Western blot showed an increase in phosphorylation for PI3K, Akt, and mTOR in cells treated with 10 ng/ml or 100 ng/ml of IL-18 compared to control. On the other hand, lower concentrations of IL-18 (1 ng/ml) did not elicit an increase in protein phosphorylation (Figure 6). These results suggested that IL-18 treatment *in vitro* stimulated the PI3K/Akt/mTOR pathway. Thus, we chose 100 ng/ml as the concentration of IL-18 for subsequent inhibition and activation experiments.

### Chondrocyte-specific gene degradation caused by IL-18 stimulation was associated with the activation of PI3K/Akt/mTOR pathway

**Figure 7:**
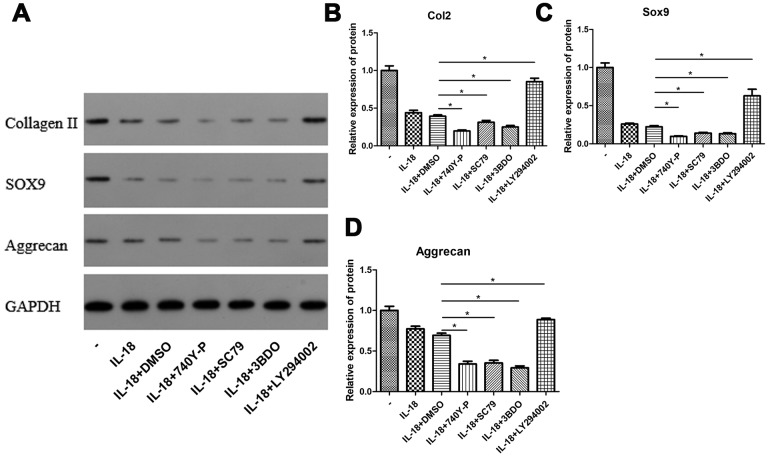
**PI3K/Akt/mTOR pathway activation was associated with the chondrocyte-specific degradation caused by IL-18 stimulation.** Chondrocytes of the IL-18 + 30 μM 740Y-P solution (or 14 μM SC79 solution or 120 μM 3BDO solution or 50 μM LY294002 solution or DMSO) treatment group were pre-treated with 740Y-P (or SC79 or 3BDO or LY294002 or DMSO) for 1 h, followed with 24 h IL-18 stimulation (100 ng/ml). The inhibitors and activator were all dissolved in DMSO, and the concentration of DMSO in all experimental groups was consistently lower than 0.1%. Protein levels of Collagen II (**B**), Sox9 (**C**), Aggrecan (**D**), and GAPDH as an internal control, evaluated by Western blot (**A**). The values are expressed as mean ± standard deviation (SD). Significance was calculated by a one-way ANOVA with a post hoc Tukey's multiple comparisons test. *p<0.05 versus the IL-18 + DMSO treatment group.

We performed a series of pathway inhibition and activation experiments to test whether activation of the PI3K/Akt/mTOR pathway contributed to the chondrocyte-specific gene degradation caused by IL-18 stimulation. IL-18-stimulated chondrocytes were treated with 740Y-P (a PI3K activator) or SC79 (an Akt activator) or 3BDO (an mTOR activator) or LY294002 (a PI3K inhibitor), and the protein levels of collagen II, sox9, and aggrecan were measured by Western blot. As shown in Figure 7, the chondrocyte-specific gene degradation caused by IL-18 stimulation was further aggravated by 740Y-P or SC79 or 3BDO treatment while LY294002 treatment ameliorated it. These results confirmed that activation of the PI3K/Akt/mTOR pathway contributes to the chondrocyte-specific gene degradation induced by IL-18 stimulation.

### Joint destruction caused by IL-18 stimulation was reduced by the treatment of rapamycin *in vivo*

**Figure 8:**
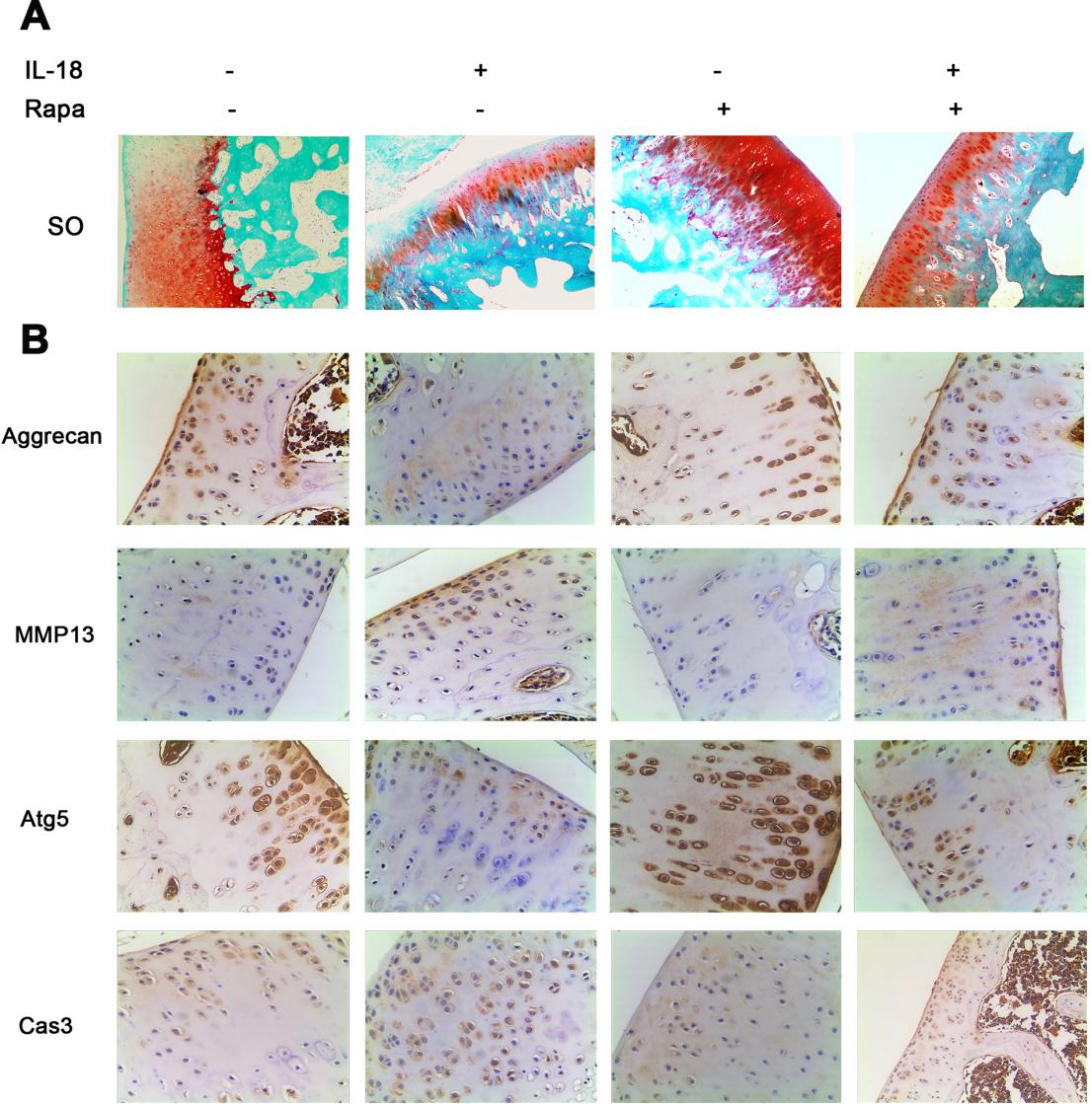
**Rapamycin showed a cartilage-protective role in a rat model.** A total of 60 SD rats were randomly divided into four groups: 15 rats in the IL-18 treatment group which received 50 μl IL-18 solution (100 ng/ml); 15 rats in the rapamycin treatment group which received 50 μl rapamycin solution (100 nM); 15 rats in the IL-18 + rapamycin treatment group which received 50 μl IL-18 + rapamycin solution (100 ng/ml and 100 nM respectively); 15 rats in the control group which received an equal volume of vehicle. (**A**) Rats were euthanized after 8 weeks of treatment, and the knees were preserved in 4% paraformaldehyde solution. 4% paraformaldehyde-fixed knees were decalcified and embedded in paraffin, then sectioned at 5 μm thickness. Sections of the interior joint were stained with safranin O-fast green (SO). (**B**) Aggrecan, MMP13, Atg5, and Caspase3 were detected by immunohistochemistry.

Rapamycin works as a potent antagonist of mTOR signaling. Here we analyzed its effect on articular cartilage integrity *in vivo*. We used safranin O-fast green (SO) staining and immunohistochemistry to evaluate the development of OA. The results of SO revealed that the knees of rats in the IL-18 treatment group showed more disrupted cartilage surface than the control group, whereas the knees of rats in the IL-18 + rapamycin treatment group showed healthier cartilage than the IL-18-only treatment group (Figure 8A). Immunohistochemistry data (Aggrecan, MMP13, Atg5 and Caspase3, Figure 8B) also showed that rapamycin treatment alleviated IL-18-induced chondrocyte-specific protein degradation, decreased the levels of inflammatory factors, enhanced autophagy, and reduced apoptosis in chondrocytes. Taken together, these results demonstrate that rapamycin treatment protects cartilage integrity in a rat model.

## DISCUSSION

As a member of the IL-1 family, IL-18 is mainly produced by chondrocytes, osteoblasts, and macrophages in joints [12–14]. Previous studies reported elevated IL-18 levels in the plasma, synovial fluid, and articular cartilage of OA patients, which correlated with radiographic severity [16, 23]. Furthermore, IL-18 exerts proinflammatory and catabolic effects in cellular and animal model chondrocytes [13, 15, 24, 25]. In addition, IL-18 may also promote chondrocyte apoptosis [20]; however, the specific underlying mechanisms remain unclear. In the present study, we observed a dose-dependent downregulation of Collagen II, Sox9, and Aggrecan caused by IL-18 stimulation, thereby demonstrating that IL-18 has catabolic effects.

Apoptosis is necessary for normal cell turnover, proper development, and chemically-induced cell death [26]. Consequently, abnormal apoptosis promotes multiple human disorders such as neurodegenerative diseases, skeletomuscular degeneration, and many types of cancer [26]. Caspases promote apoptosis, and their activation and function is regulated by the Bcl-2 family of proteins, among other molecules [27, 28]. The Bcl-2 family consist primarily of three factions: the anti-apoptotic subfamily (including Bcl-2 and Bcl-xl) and two pro-apoptotic factions, the BH3-only proteins (such as BIM and BID), which convey various cytotoxic signals, and the death effectors Bax and Bak [29]. Our study demonstrated that pro-apoptotic genes such as Bax, Caspase3, and Caspase9 are upregulated upon IL-18 treatment while the anti-apoptotic gene Bcl2 is downregulated. This result indicated that IL-18 promotes apoptosis in rat chondrocytes *in vitro*, which is consistent with a previous study on human articular chondrocytes by John T et al. Furthermore, our SA-β-Gal assays showed that IL-18 treatment increased cellular senescence, which might contribute to the destruction of articular cartilage.

Autophagy is a cellular homeostatic and survival mechanism involving catabolic processes in eukaryotic cells [30, 31]. Its dysregulation is associated with several age-related diseases, including degenerative OA [31]. Indeed, while autophagy helps to maintain cellular homeostasis in normal human articular cartilage, autophagy declines with aging and during OA development. Recent studies have shown that autophagy engages in a complex crosstalk with apoptosis [32–34]. Caramés B et al reported that reduced autophagy is often accompanied by increased apoptosis and cartilage degeneration [34]. Caroline B et al suggested that there is a transient increase in autophagy during early OA as a compensatory response to cellular stress and that apoptosis ensues when prolonged stress overwhelms this mechanism [35]. In this study, we investigated the effects of IL-18 treatment on chondrocyte autophagy. Our results consistently showed that IL-18 treatment could transiently increase autophagy, which then decreased over time. We also found an accumulation of P62 protein in chondrocytes, implying that IL-18 suppressed autophagic degradation. Such results showed that IL-18 stimulation caused an autophagy deficiency in chondrocytes, which may promote chondrocyte apoptosis and degradation.

The main regulator of autophagy in chondrocytes is mTOR, a serine/threonine kinase [36], which forms two distinct signaling complexes, mTOR complex 1 (mTORC1) and mTORC2 [22]. The former is a negative regulator of autophagy with the PI3K/Akt pathway being its main upstream modulator [37]. Several studies have demonstrated that inhibition of the PI3K/Akt/mTOR signaling pathway promotes autophagy in chondrocytes and elicits chondro-protective effects in OA [38–40]. In our study, we found that rapamycin, a selective mTOR inhibitor, increased the levels of autophagy-related proteins Atg7 and LC3, which were suppressed by IL-18 stimulation. Inhibition of such proteins promoted IL-18-induced apoptosis, suggesting that autophagy inhibition might be an early step in IL-18-induced apoptosis in chondrocytes. In addition, we showed that IL-18 activated the PI3K/Akt/mTOR signaling pathway in a dose-dependent manner (Figure 6). In order to confirm the connection between the PI3K/Akt/mTOR signaling pathway and IL-18-induced chondrocyte degradation, we carried out various pathway activation and inhibition experiments. Our results showed that 740Y-P (a PI3K activator) or SC79 (an Akt activator) or 3BDO (an mTOR activator) treatment further decreased Collagen II, Sox9, and Aggrecan levels whereas LY294002 (a PI3K inhibitor) treatment increased Collagen II, Sox9, and Aggrecan levels in the presence of IL-18. These results indicated that the PI3K/Akt/mTOR signaling pathway is activated in IL-18-induced chondrocyte degradation and that its inhibition correspondingly elicits chondro-protective effects by restoring autophagy.

We also tested whether IL-18-induced cartilage degeneration could be rescued *in vivo* by inhibiting the PI3K/Akt/mTOR signaling pathway. To this end, we used a rat model to evaluate the effects of rapamycin treatment on cartilage degeneration through SO staining and immunohistochemistry experiments. Our results showed that rapamycin exerts a protective effect on cartilage in rats. In conclusion, our results demonstrated that activation of the PI3K/Akt/mTOR signaling pathway promoted the pro-apoptosis effects of IL-18 treatment in rat chondrocytes *in vitro* and *in vivo* while its inhibition promoted autophagy with protective effects on chondrocytes. These findings further our understanding of IL-18-induced apoptosis in chondrocytes and highlight IL-18 and the mTOR pathway as potential therapeutic targets in the treatment of cartilage degeneration in OA.

## MATERIALS AND METHODS

### Materials

Recombinant rat IL-18, rat 740Y-P as PI3K activator, rat SC79 as AKT activator, rat 3BDO as mTOR activator, and rat LY294002 as PI3K inhibitor were purchased from R&D Systems, Abingdon, UK. Dulbecco’s Modified Eagle’s Medium (DMEM), fetal bovine serum (FBS), penicillin and streptomycin, 0.25% trypsin were obtained from Gibco RRL, Grand Island, NY, USA. Collagenase II came from Sigma-Aldrich, St, Louis, MO, USA.

### Cell culture and treatment

Primary cartilage pieces isolated from the knee joints of four-week-old Sprague Dawley rats was digested with 0.2% collagenase II on a horizontal shaker for 4 h at 37 °C. Then chondrocytes were harvested and grown in DMEM containing 10% FBS and 1% penicillin/streptomycin at 37 °C with 5% CO2. Cells were seeded in six-well plates for analysis. Subconfluent cells were treated with different concentrations of IL-18 (0 ng/ml as control, 1 ng/ml, 10 ng/ml, 100 ng/ml) for 24 h, and then cells were processed to assess the effects of IL-18 on chondrocyte-specific proteins and matrix-degradation, apoptosis, senescence, and autophagy. Based on the results from such initial experiments, we chose 100 ng/ml as the concentration of IL-18 for subsequent pathway activation and inhibition tests. In pathway activation and inhibition tests, subconfluent cells were pretreated with 740Y-P (30 μM) or SC79 (14 μM) or 3BDO (120 μM) or LY294002 (50 μM) for 1 h then and then incubated with IL-18 (100 ng/ml) for 24 h before analysis. This study was approved by the Ethics Committee of the 2^nd^ Affiliated Hospital, School of medicine, Zhejiang University, Hangzhou, China.

### RNA extraction and real-time PCR

Total RNA from chondrocytes was extracted by using TRIzol reagent (Invitrogen, Carlsbad, CA, USA) according to the manufacturer’s instructions. Total RNA was used to synthesize cDNA by reverse transcription (cDNA synthesis kit, Takara). Power SYBR Green PCR Master Mix (Applied Biosystems) was used for real-time PCR. The mRNA levels of collagen II, sox9, and aggrecan were analyzed using primer sequences listed in Table 1. For real-time PCR, 10 μL reaction mixture containing SYBR Green and each primer was used. PCR reactions were carried out in circulations, pre-denaturation at 95 °C for 1 min and 40 circulations of denaturation at 95 °C for 15 s, primer annealing, and extension at 63 °C for 25 s, followed by melt curve analysis. The GAPDH gene was used as the reference gene. Data were analyzed for fold difference using the following formula: 2^(CT GAPDH-CT targeted gene)^ ×10.

**Table 1:** Primers used for real-time PCR.

**Gene**	**Forward**	**Reverse**
Rat Collagen II	GAGTGGAAGAGCGGAGACTACTG	GTCTCCATGTTGCAGAAGACTTTCA
Rat Sox9	CCAGCAAGAACAAGCCACAC	CTTGCCCAGAGTCTTGCTGA
Rat Aggrecan	CTAGCTGCTTAGCAGGGATAACG	GATGACCCGCAGAGTCACAAAG
Rat GAPDH	GAAGGTCGGTGTGAACGGATTTG	CATGTAGACCATGTAGTTGAGGTCA

### Western blot

Total cellular proteins were extracted with RIPA containing protease and phosphatases inhibitors according to the manufacturer’s protocol. Equal amounts of extracted proteins were separated by 10% SDS-PAGE electrophoresis, and wet transmembrane (PVDF membrane) was conducted for 2 h. After being blocked at room temperature for 1 h with 5% nonfat milk, membranes were incubated with primary antibodies (purchased from Sigma company) against GAPDH (Abcam ab181602), Collagen II (Abcam ab34712), Sox9 (Abcam ab185230), Aggrecan (Biorbyt orb10066), Bax (Abcam ab32503), Bcl2 (Abcam ab194583), Cleaved Caspase3 (CST 9661), Pro-Caspase3 (Abcam ab9043), Cleaved Caspase9 (CST 9501), Pro-Caspase9 (Abcam ab2013), Atg5 (Abcam ab78073), Atg7 (Proteintech 10088-2-AP), Beclin1 (Abcam ab62557), LC3B (CST 4108), PI3K (Abcam ab86714), p-PI3K (Abcam ab182651), Akt (CST 4691), p-Akt (CST 4060), mTOR (CST 5536), and p-mTOR (CST 2983) overnight at 4 °C. Afterwards, the membranes were washed with TBST three times for 10 min and then incubated with secondary antibodies at room temperature for 1 h. Finally, signals were detected using West Dura Extended Duration Substrate with exposure to X-ray film.

### Immunofluorescence

Chondrocytes cultured on 24-well plates were incubated with IL-18 for varying times (0, 6, 24 h). After fixation with methanol for 30 min, the cells were permeabilized by PBS containing 0.5% v/v Triton X-100 for 15 min and blocked with 5% BSA for 1 h. The chondrocytes were incubated with primary antibody against LC3B at 4 °C overnight. After that, the chondrocytes were incubated with fluorescein isothiocyanate-conjugated secondary antibodies for 1 h. Cell nucleuses were stained with DAPI for 5 min. Finally, the cells were analyzed with a Leica fluorescence microscope.

### Senescence associated β-galactosidase (SA-β-Gal) assay

Cytochemical staining for SA-β-Gal was performed using a SA-β-Gal staining kit. Briefly, chondrocytes in primary culture were seeded onto 6-well culture plates at density of 1×10^5^ cells/well and cultured for 24 h at 37 °C in an incubator under 20% O_2_/5% CO_2_. Then chondrocytes were treated with different concentrations of IL-18 (0, 1, 10, 100 ng/ml). Cytochemical staining for SA-β-Gal was performed at pH 6 according to the manufacturer’s protocol 24 hours after IL-18 treatment, and the positive cells in four randomly selected fields per treatment were counted. The experiment was repeated three times.

### Animal experiments

Total 60 SD rats (200-250 g; 6 weeks old) were randomly divided into four groups (15 rats in each group). For the IL-18 treatment group, 50 μl IL-18 solution (100 ng/ml) were administered by intra-articular injection twice a week; for the rapamycin treatment group, 50 μl rapamycin solution (100 nM) was injected intra-articularly twice a week; for the IL-18 + rapamycin treatment group, 50 μl IL-18 + rapamycin solution (100 ng/ml and 100 nM respectively) was injected intra-articularly twice a week; for the control group, an equal volume of vehicle was used. All rats were euthanized after 8 weeks of treatment, and the knees were preserved in 4% paraformaldehyde solution.

### Histological analysis

4% paraformaldehyde solution fixed knees were decalcified and then embedded in paraffin, followed by sectioning at 5 μm thickness. The sections of interior joint were stained with SO.

### Immunohistochemical analysis

Sections were immunohistochemically stained for assessment of OA. Ab against Aggrecan (Abcam, ab36861), MMP13 (Abcam, ab219620), Atg5 (Abcam, ab108327) and Caspase3 (Abcam, ab4051) were used in this analysis.

### Statistical analysis

Statistical analyses were conducted with software SPSS version 12.0. We calculated the means and standard deviations for all data. Differences between groups were analyzed using One-way ANOVA with a subsequent *post hoc* Tukey’s test. P<0.05 is considered statistically significant.

## Supplementary Material

Supplementary Figures
