# Supplementary material for: Rapamycin protects chondrocytes against IL-18-induced apoptosis and ameliorates rat osteoarthritis
Source: Aging (Albany NY). 2020 Mar 17;12(6):5152–67. doi: 10.18632/aging.102937 (PMC7138594; doi:10.18632/aging.102937)
Supplement: Supplementary Figures [file aging-12-102937-s001..pdf]

SUPPLEMENTARY FIGURES

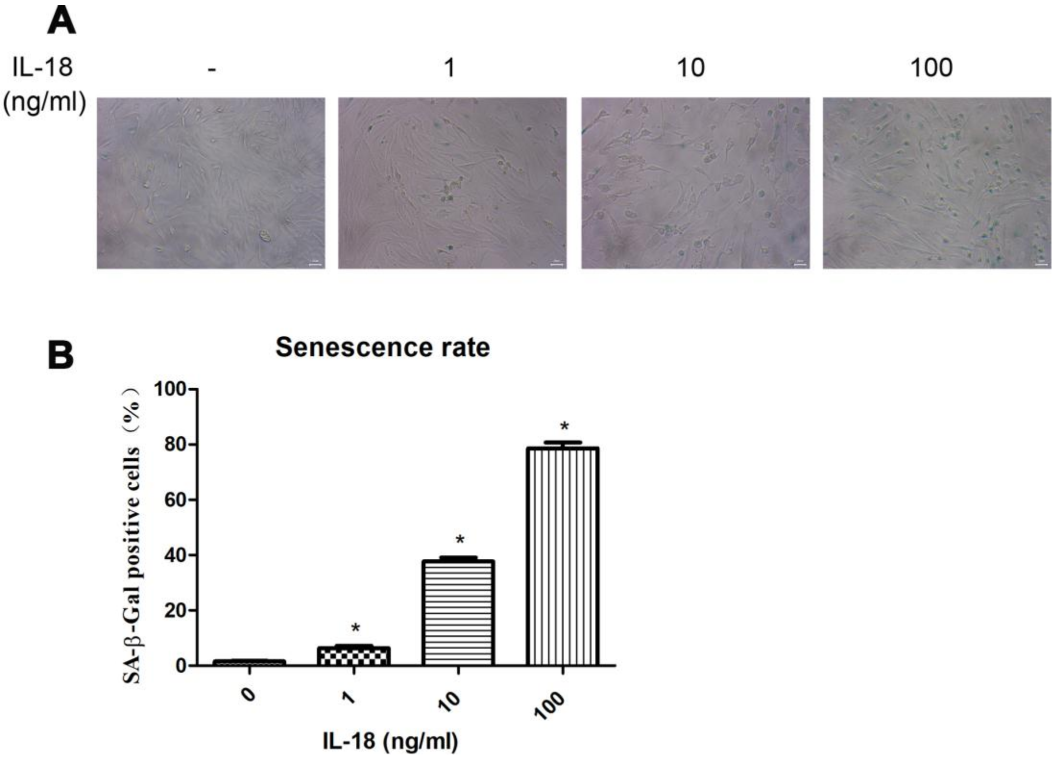

Supplementary Figure 1. IL-18 stimulation increased the senescence rate of normal rat chondrocytes. The chondrocytes were treated with IL-18 at different concentrations for 24 h. The SA-β-Gal positive cells (**A**) were observed under a microscope (Scale bar = 20 μm), and the percentage of SA-β-Gal positive cells (**B**) was calculated (n=3). The values are expressed as mean ± standard deviation (SD). Significance was calculated by a one-way ANOVA with a *post hoc* Tukey's multiple comparisons test. *p<0.05 versus 0 ng/ml IL-18 treated group.

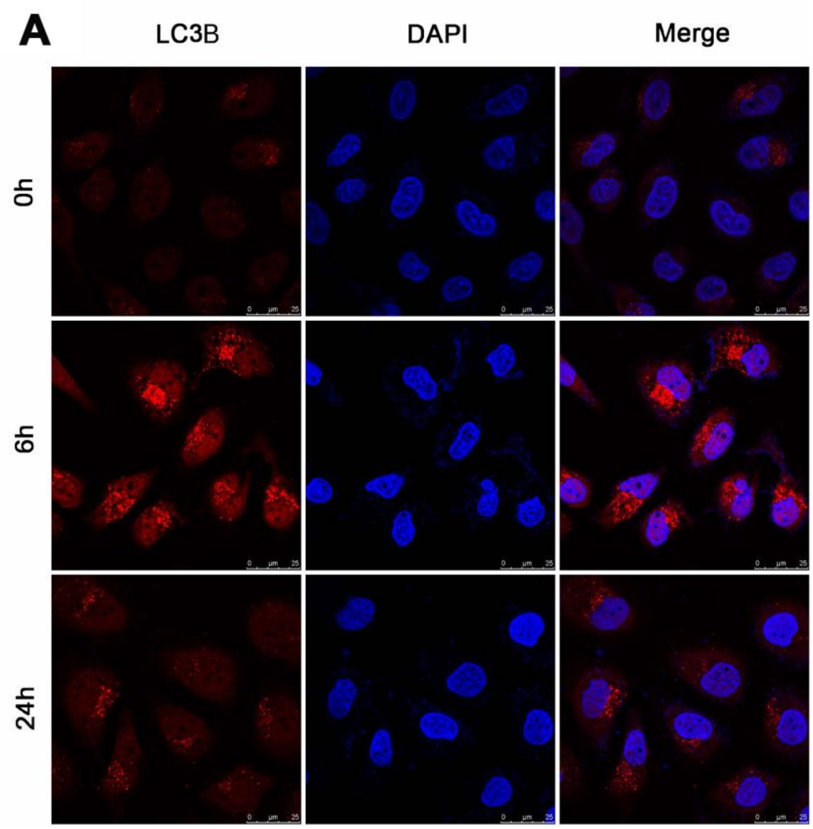

Supplementary Figure 2. IL-18 stimulation repressed the autophagy of rat chondrocytes. The chondrocytes were incubated without or with IL-18 (100 ng/ml) for various durations. Immunofluorescence of LC3B in treated cells (red: LC3B; blue: DAPI) was evaluated (**A**). Bar = 25 μm.
